# The effect of BMP9 on inflammation in the early stage of pulpitis

**DOI:** 10.1590/1678-7757-2022-0313

**Published:** 2023-01-23

**Authors:** Tianzhu SONG, LI Xiangfen, LIU Liu, Yanglin ZENG, Dongzhe SONG, Dingming HUANG

**Affiliations:** 1 Sichuan University West China Hospital of Stomatology State Key Laboratory of Oral Diseases Chengdu China Sichuan University, West China Hospital of Stomatology, State Key Laboratory of Oral Diseases and National Clinical Research Center for Oral Diseases, Chengdu, China.; 2 Northwest Minzu University Key Laboratory of Stomatology of State Ethnic Affairs Commission Key Laboratory of Oral Diseases of Gansu Province Lanzhou Gansu China Northwest Minzu University, Key Laboratory of Stomatology of State Ethnic Affairs Commission, Key Laboratory of Oral Diseases of Gansu Province, Lanzhou, Gansu, China.; 3 Sichuan University West China Hospital of Stomatology Jinjiang District Out Patient Section Chengdu China Sichuan University, West China Hospital of Stomatology, Jinjiang District Out Patient Section, Chengdu, China.; 4 Sichuan University West China Hospital of Stomatology Department of Conservative Dentistry and Endodontics Chengdu China Sichuan University, West China Hospital of Stomatology, Department of Conservative Dentistry and Endodontics, Chengdu, China.

**Keywords:** Growth Differentiation Factor 2, Pulpitis, Dental Pulp, Wound Healing

## Abstract

Bone morphogenetic protein 9 (BMP9) tends to be associated with various inflammatory responses of diseases, but its relationship with pulpitis remains unknown.

**Objective:**

This study aimed to evaluate the effects and mechanisms of BMP9 in pulpitis.

**Methodology:**

A rat model of pulpitis was used to evaluate the expression of BMP9, which was also analysed in Porphyromonas gingivalis lipopolysaccharide (*Pg*-LPS)-stimulated human dental pulp cells (hDPCs). The effects and mechanism of BMP9 on the regulation of inflammatory factors and matrix metalloproteinase-2 (MMP2) were evaluated using real-time quantitative PCR, western blotting, and immunocytofluorescence. Moreover, the migration ability of THP-1 monocyte-macrophages, treated with inflammatory supernate inhibited by BMP9, was previously tested by a transwell migration assay. Finally, a direct rat pulp capping model was used to evaluate in vivo the influence of the overexpression of BMP9 in pulpitis.

**Results:**

The expression of BMP9 decreased after 24 h and increased after 3 and 7 d in rat pulpitis and inflammatory hDPCs. The overexpression of BMP9 inhibited the gene expression of inflammatory factors (IL-6, IL-8, and CCL2) and the secretion of IL-6 and MMP2 in *Pg*-LPS-stimulated hDPCs. The level of phosphorylated Smad1/5 was upregulated and the levels of phosphorylated ERK and JNK were downregulated. The inflammatory supernate of hDPCs inhibited by BMP9 reduced the migration of THP-1 cells. In rat pulp capping models, overexpressed BMP9 could partially restrain the development of dental pulp inflammation.

**Conclusion:**

This is the first study to confirm that BMP9 is involved in the occurrence and development of pulpitis and can partially inhibit its severity in the early stage. These findings provided a theoretical reference for future studies on the mechanism of pulpitis and application of bioactive molecules in vital pulp therapy.

## Introduction

Pulpitis is a common disease that affects dental health. Without control, it can lead to infection of the entire pulp and even periapical inflammation. The effective control of pulpitis while preserving a healthy pulp tissue is a challenge for dentists. Vital pulp therapy (VPT) could be a solution, as it covers the infected pulp with biocompatible materials to promote pulp tissue repair, preserving the healthy pulp tissue and the normal physiological function of teeth.^1^ However, there is no exact technology to completely remove the infected pulp, especially for teeth with an ambiguous pulpitis status or relatively serious pulp inflammation, which ultimately leads to treatment failure.^2^ Therefore, analysing the key factors that regulate pulpitis and promote pulp tissue repair and combining them with bioactive materials can be a potential solution for this problem and provide a reference to develop new bioactive molecular materials.^3^

Bone morphogenetic protein 9 (BMP9) is a unique member of the BMP family and closely related to tooth development. In adults, it is produced mainly in the liver and enters the blood to function in a biologically active state.^4,5^ BMP9 has strong osteogenic potential and is deeply involved in metabolism, vascular homeostasis, and tumorigenesis or tumor inhibition.^6-9^ The relationship between BMP9 and teeth has been studied more recently. BMP9 knockout mice had teeth dysplasia.^10^ *In vitro* experiments showed that BMP9 can promote the odontogenic and osteogenic differentiation of dental papilla and periodontal membrane cells.^11,12^ Compared with other members of the BMP family, there are few studies on the role of BMP9 in oral tissue diseases. Studies showed that BMP9 has a stronger osteogenic ability compared with BMP2/7. Our research team has previously evaluated the ability of BMP9 to promote dentin formation in dental pulp tissue under direct pulp capping. Our results showed that BMP9 could be expressed in the odontoblast cell layer and central region of human pulp tissue and has a powerful function of promoting pulp injury repair in a rat pulp capping model.^13^ Moreover, BMP9 may be a potential bioactive factor for the development of new bioactive molecular materials for VPT. However, more comprehensive studies are needed, especially to assess changes in the expression of BMP9 and its regulatory effects in pulpitis, since our and other similar pulp capping experiments were performed in an inflammatory environment. We only focused on the role of biological factors in promoting pulp tissue repair, ignoring whether they also play a regulatory role in pulp tissue inflammation.

BMP9 is associated with inflammation in various tissue diseases, including vascular diseases, liver fibrosis, osteoarthritis, and cancer inflammation. After treating hepatic stellate cells and vascular endothelial cells with BMP9, the levels of inflammatory factors (IL-6, IL-8, and TNF-α), chemokines, and inflammatory signaling pathways significantly changed.^14^ BMP9 also reduced cancer metastasis by inhibiting the inflammatory microenvironment.^15,16^ Currently, there are few studies on BMP9 and dental inflammatory diseases. TNF-α is released in the periapical inflammatory region and BMP9 can partially restore the inhibitory effect of TNF-α on osteoblast differentiation of dental papilla cells.^17^ No studies evaluate BMP9 and dental pulp inflammation, however, the correlation between BMP9 and pulpitis requires further discussion, which will provide not only a basis for understanding the molecular regulatory mechanism in the occurrence and development of pulpitis, but a more comprehensive basic research reference for BMP9 as a biological factor in new bioactive molecular pulp capping materials. A comprehensive understanding of the molecular regulatory mechanism of pulpitis is also the basis for identifying effective inflammatory regulatory factors in new VPT pulp capping materials. Therefore, this study was the first to analyse the expression pattern of BMP9 in the occurrence and development of pulpitis and its regulatory effect on this condition to provide a theoretical basis for evaluating bioactive molecules suitable for VPT.

## Methodology

### Ethics statement

In this study, the use of human samples and rats was approved by the Stomatology Ethics Committee and the State Key Laboratory of Oral Diseases (WCHSIRB-D-2020-368). All teeth were collected after patients signed the informed consent form.

### Rat model of pulpitis

The use of rat models of pulpitis followed methods from previous studies.^18^ According to the sample collection time points, after opening the pulp chamber and exposing pulp tissue, 20 male Sprague-Dawley (SD) rats (eight weeks old, weighting about 220 g) were randomly divided into four groups: day 1, day 3, day 7, and control group (untreated). Rats underwent general intraperitoneal anesthesia (10% chloral hydrate, J&K Scientific, Beijing, China). A cavity was made on the occlusal plane of the mandibular first molars of rats with a spherical burr (size 1/4) (Toboom, Shanghai, China) at high speed without cooling. After anesthesia, rats were euthanized by cervical dislocation at different time points and mandibular tissues were immediately collected and fixed in 4% paraformaldehyde (Biosharp, Beijing, China) for 2 d.

### Direct rat pulp capping models

Based on previous studies,^19,20^ 15 SD rats were randomly divided into three groups and intraperitoneal anesthesia was performed with 10% chloral hydrate. The maxillary first molar surface of rats was cleaned and disinfected with 75% alcohol (Ausbebo, Shandong, China). Then, using a sterilized high-speed 1/4 ball drill and assisted cooling with normal saline, a class I cavity was made on the occlusal surface of the maxillary first molar of rats. Finally, the pulp was opened with a sterile #30 K-File (Dentsply Sirona, Charlotte, NC, USA). The three groups were treated with HCl, lipopolysaccharide (LPS)+HCl, and LPS+BMP9 (rhBMP9, R&D Systems, Minneapolis, MN, USA), respectively. The BMP9 recombinant protein was reconstituted with 4 mM of HCl (Codow, Guangdong, China). Sterile gelatin sponges (GSs) (HU SHI DA, Nanchang, China) soaked in *Porphyromonas gingivalis* lipopolysaccharide (*Pg*-LPS) (10 mg/mL; Invivogen, San Diego, CA, USA) were placed on the exposed pulp of rats treated with LPS+HCl and LPS+BMP9. GSs soaked in PBS (Beyotime, Shanghai, China) were placed on the exposed pulp of rats treated with HCl. All GSs were removed after 30 min. GSs soaked in BMP9 were placed on the exposed pulp of rats treated with LPS+BMP9 and GSs soaked in HCl were placed on the exposed pulp of rats of the other two groups. Later, the cavity was perfectly sealed with conventional glass ionomer cement (New Century Dental, Shanghai, China). Rats were euthanized by cervical dislocation 24 h after anaesthesia and maxillary samples were immediately collected and fixed in 4% paraformaldehyde for 2 d.

### Immunohistochemical (IHC) staining

The tissue fixed with paraformaldehyde was demineralized with 10% EDTA (J&K Scientific) for one month, embedded in paraffin, and cut into 5-µm sections. Paraffin sections were dewaxed in xylene (J&K Scientific), rehydrated with distilled water, and subjected to antigen retrieval with sodium citrate (Solarbio) for 15 min at 95°C or proteinase K (Solarbio) for 15 min at 37°C. Standardized IHC kits (Zhongshan Jinqiao, Beijing, China) were used. Samples were incubated overnight at 4°C with the following antibodies: BMP9 (1:800; R&D Systems), IL-6 (1:400; Abcam, Cambridge, UK), and IL-8 (1:400; Novus Biologicals, Centennial, CO, USA).

### Cell culture

In accordance with previous studies,^21^ pulp tissues from orthodontic teeth or third molars were extracted from patients younger than 20 years old. Human dental pulp cells (hDPCs) were isolated and cultured in an α-modified Eagle’s medium (Gibco, Carlsbad, CA, USA) supplemented with 10% fetal bovine serum (Gibco), 50 mg/mL of streptomycin, and 100 U/mL of penicillin (Sigma-Aldrich, St. Louis, MO, USA) at 37°C in a humidified atmosphere with 5% CO_2_. Cells were passaged when they reached about 80% confluence. Passages 3 to 5 of hDPCs were used in subsequent experiments.

THP-1 cells are human acute monocytic leukemia cell lines that can be transformed into adherent macrophages induced by 12-O-tetradecanoylphorbol-13-acetate 9 (TPA) *in vitro*. They are mainly used to study monocyte-macrophages and their immune regulation function. THP-1 cells (Cell Bank of Type Culture Collection of Chinese Academy of Sciences, Shanghai, China) were seeded in a T25 flask (Corning, NY, USA) with a RPMI-1640 medium (Gibco) containing 10% FBS and 1% double antibody at 37°C and 5% CO_2_. When the cell culture medium turned yellow (after about 3 d), the fluid with half of the volume was changed or cell passage was performed. TPA (100 ng/mL) (APExBIO, Houston, TX, USA) was used to induce THP-1 cells to differentiate into adherent macrophages and cells adhered to the wall after 48 h.

### Recombinant adenoviruses expressing BMP9 and the green fluorescent protein (GFP)

Recombinant adenoviruses were generated using AdEasy, in line with previous studies, which used it to change the gene expression in hDPCs.^22,23^ In this study, hDPCs were infected with recombinant adenoviruses expressing GFP (Ad-GFP) and BMP9 (Ad-BMP9). Ad-BMP9 also expresses GFP as a marker to monitor infection efficiency. Ad-GFP expressing only GFP was used as control. Both fluorescence level and qPCR showed changes in the gene expression of BMP9. After hDPCs were seeded in culture plates or dishes, the adenovirus was added to the culture medium to infect cells and the expression of BMP9 reached a high and stable level after 48 h. At this moment, *Pg*-LPS was added to the culture medium. This time point was zero hours of *Pg*-LPS stimulation.

### *Pg*-LPS stimulation

LPS in this study derived from *Porphyromonas gingivalis. Pg*-LPS (InvivoGen) was added to stimulate hDPCs to produce an inflammatory response. The optimal amount of *Pg*-LPS was determined by CKK8 (Beyotime) and the expression of inflammatory cytokines after stimulation. *In vitro* experiments were performed in this study using three treatment groups: GFP, GFP+LPS, and BMP9+LPS.

### Real-time quantitative PCR analysis

In this study, hDPCs were seeded in 6-well plates and RNA was extracted after 3, 6, and 12 h after the addition of *Pg*-LPS. The effect of BMP9 alone on inflammatory factors was also tested. Total RNA from the 6-well plate cells was isolated using the TRIzol^TM^ reagent (Invitrogen), according to the manufacturer’s instructions. Extracted RNA products were retrotranscribed and quantitatively detected (Takara, Tokyo, Japan). Glyceraldehyde-3-phosphate dehydrogenase (GAPDH) was used as control. Primers were used to generate products of about 200 bp for an efficient analysis. Primer sequences are listed in Supplementary Table S1.

### ELISA

After confirming changes in the expression of genes encoding inflammatory factors, changes at the protein level (represented by IL-6) were evaluated. The IL-6 level in the culture medium was measured using ELISA (Signalway Antibody, College Park, MD, USA). The cell culture supernatant was centrifuged at 3000 RPMs for 10 min to remove particles and polymers. After treatment with standard operating procedures, the absorbance was measured using a microplate reader at a wavelength of 450 nm. In this study, hDPCs were used in western blotting (WB) to detect the expression of matrix metalloproteinase-2 (MMP2) related to inflammation.

### Transwell migration assay

Immune cells play an important role in inflammation. Therefore, the effect of the supernatant of inflammatory hDPCs subjected to different treatments on the migration ability of THP-1 cells was evaluated *in vitro* in a transwell chamber. In this study, hDPCs were divided into three treatment groups: GFP, GFP+LPS, and BMP9+LPS. After 48 h of virus infection, according to the aforementioned virus infection method, the medium was replaced by a serum-free medium and *Pg*-LPS was added. The supernatant of different experimental groups was collected and centrifuged at 3000 RPMs for 5 min to remove cell fragments 24 h after the addition of *Pg*-LPS. A sample of 5×10^5^ THP-1 cells was inoculated into 6-well plates with 2 mL of cells in each and TPA was added to induce cell adherence. After 48 h, cells were digested with trypsin (Gibco) and counted to 1×10^6^. Later, 200 μL of cell suspension and 600 μL of the collected supernatant were added to the upper and lower transwell chamber (Corning), respectively. After 24 h, cells in the upper chamber were removed, fixed with paraformaldehyde, and stained with 0.5% crystal violet (Solarbio Science & Technology Co. Ltd., Beijing, China). Images were taken by a microscope and the number of migrating cells was calculated for statistical analysis.

### WB analysis

Cell lysates (Signalway Antibody) containing phosphatases and protease inhibitors were extracted and whole-cell proteins were extracted from 6-cm dishes. The protein concentration was measured using a BCA protein assay kit (Thermo Fisher Scientific, Waltham, MA, USA). Denatured protein samples (20–40 μg) were loaded onto a 10% polyacrylamide gel (Beyotime) and separated using electrophoresis. A PVDF membrane (Millipore, Billerica, MA, USA) was used for protein transfer and 5% skim milk powder (Solarbio) or bovine serum albumin (BSA) (Solarbio) was used for antigen blocking. Then, the membrane was incubated overnight at 4°C with primary antibodies, including anti-BMP9 (sc-514211; 1:400; Santa Cruz Biotechnology, Dallas, TX, USA), anti-MMP2 (29090; 1:1000; Signalway Antibody), anti-MAPKs (ERK/p-ERK; p38/p-p38; JNK/p-JNK) (4695S/4370S; 8690S/4511S; 9252S/9255S; 1:1000; Cell Signaling Technology, Danvers, CO, USA), anti-p-Smad1/5/Smad1 (9516S/6944S; 1:1000; Cell Signaling Technology), and anti-GAPDH (ab8245/ab181602; 1:10000; Abcam). Secondary antibodies incubated with the membrane were rabbit anti-mouse immunoglobulin G conjugated to horseradish peroxidase (sc-2005/sc-2004; 1:10000; Santa Cruz Biotechnology). Immunoblot bands were visualized using enhanced chemiluminescence with a Bio-Rad detection system (Bio-Rad, Hercules, CA, USA).

### Immunocytofluorescence

Cells of different treatment groups in 48-well plates were fixed with paraformaldehyde after 5 h of *Pg*-LPS stimulation, permeabilized with Triton X-100 (Solarbio), blocked with 5% BSA, and incubated overnight with primary antibodies against p-ERK, p-JNK, and p-Smad1/5 (1:400; Cell Signaling Technology). The fluorescent secondary antibody (1:200; HUABIO, Hangzhou, China) was incubated with cells for 1 h and an anti-fluorescence quenching agent containing DAPI (Solarbio) was added for microscopic observation.

### Statistical analysis

All experiments were repeated at least three times. Data were expressed as mean±standard deviation. The significance was determined using Student’s t-test or an analysis of variance (ANOVA). The statistically significant value was p<0.05.

## Results

### Decreased and significantly increased expression of BMP9 after 24 h and 3 and 7 d in rats with pulpitis

**Figure 1:**
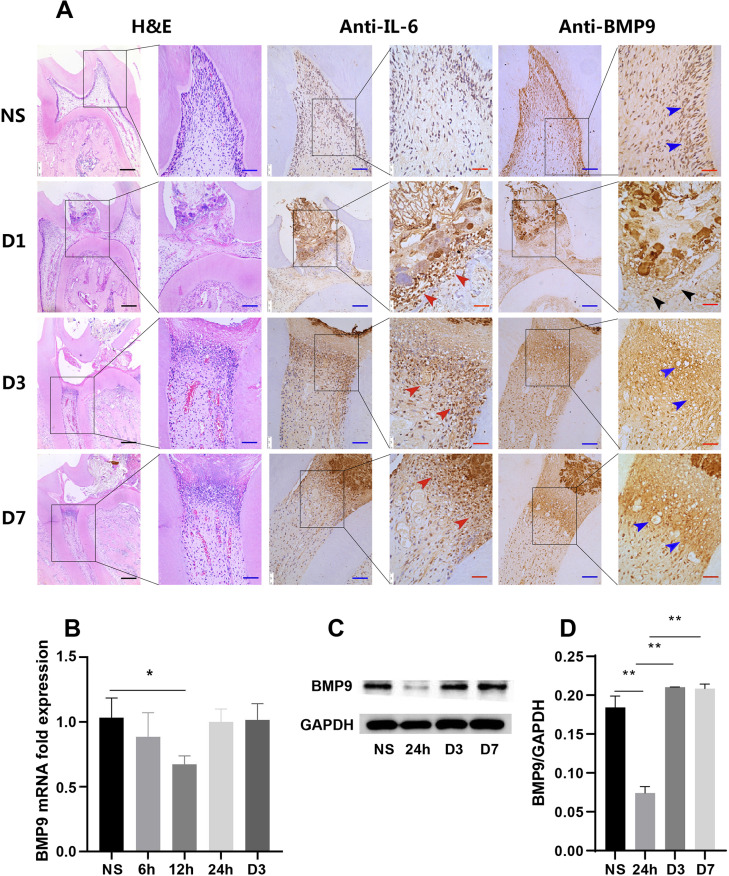
The expression of BMP9 in rat pulpitis and hDPCs. (A) H&E showed the occurrence and development of rat pulpitis at different time points; IHC showed the expression of BMP9 (blue and black arrows) and IL-6 (red arrows). (B) RT-qPCR showed that the mRNA expression of BMP9 significantly decreased in Pg-LPS-stimulated hDPCs at 12 h and then recovered. (C) WB showed that the protein levels of BMP9 decreased at 24 h, which is later than the gene decline time, in line with IHC results. (D) Quantitative analysis of WB results. NS: normal sample. Bars represent mean±SD (n=5). * p<0.05; ** p<0.01. Black scale bars: 200 μm; blue scale bars: 50 μm; red scale bars: 25 μm.

Whether BMP9 was produced or its expression changed was first analysed during the pulpitis development in rats. We successfully established a rat model of pulpitis and obtained intact pulp sections, including the crown and root, to ensure the largest area of inflammation. Hematoxylin and eosin (H&E) staining showed that 24 h after treatment, there was an acute inflammatory response below the puncture point, inflammatory cell infiltration, and odontoblast layer disorder (Figure 1 A/D1). After 3 and 7 d, the coronal pulp was necrotic, inflammatory cells were confined to the junction between the crown and the root, and the root pulp was normal (Figure 1 A/D3 and D7). We also confirmed that IL-6 was expressed in the inflammatory cell infiltration area, showing the accuracy of the inflammatory area description (Figure 1 A, red arrows). IL-6 was strongly expressed in the inflammatory area, but the high expression of IL-6 near the opening of the pulp cavity was in sharp contrast with the expression of BMP9 in this area.

BMP9 was mainly expressed in the odontoblast layer and produced in other DPCs (Figure 1 A/NS, blue arrows). However, the expression of BMP9 significantly reduced in the area of inflammation, as well as the odontoblast layer around the inflammatory area, after 24 h (Figure 1 A/D1, black arrows), which was in sharp contrast with the high expression of IL-6. After 3 and 7 d, the expression of BMP9 significantly increased in and around the inflammatory area, as well as at the margins of the inflammation (Figure 1 A/D3 and D7, blue arrows).

### Decreased expression of BMP9 after 24 h returning to normal levels in *Pg*-LPS-stimulated hDPCs after 3 d

We evaluated the expression of BMP9 in *Pg*-LPS-stimulated hDPCs *in vitro*. After detecting CCK-8 and inflammatory factor expression levels, we used 10 µg/mL of *Pg*-LPS to stimulate hDPCs *in vitro* and build an inflammatory microenvironment (Supplementary Figure S1). Our findings were in accordance with *in vivo* results. The expression of BMP9 was downregulated after 24 h and then increased (Figure 1 C and D). Moreover, results were logically consistent with changes in protein levels. The gene expression of BMP9 began to decrease after 6 h, reached its lowest level after 12 h, and then returned to normal levels (Figure 1 B).

### Regulation of BMP9 in *Pg*-LPS-induced inflammatory cytokines and MMP2 of hDPCs

**Figure 2:**
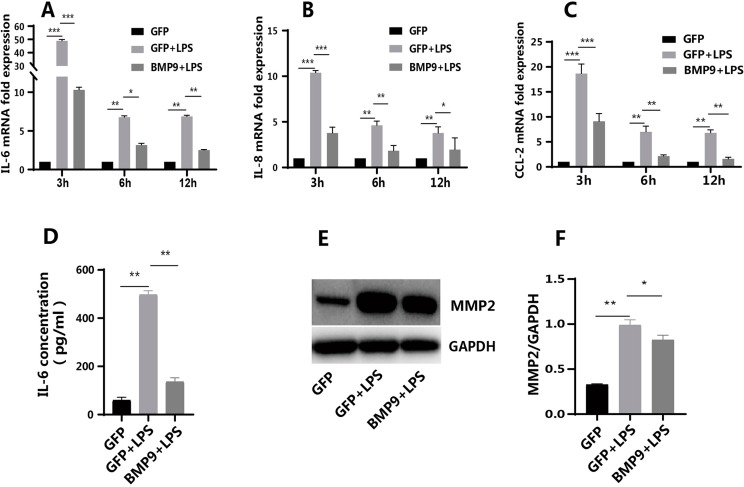
BMP9 inhibited the expression of inflammatory factors and MMP2 in hDPCs in an inflammatory state. The overexpression of BMP9 inhibited the expression of IL-6 (A), IL-8 (B), and CCL2 (C) in Pg-LPS-stimulated hDPCs at 3, 6, and 12 h (RT-qPCR). (D) ELISA confirmed that the overexpression of BMP9 reduced the expression of IL-6 at 24 h. (E) WB showed that the overexpression of BMP9 partially inhibited the expression of MMP2 at 24 h. (F) Quantitative analysis of WB results. Bars represent mean±SD (n=3). * p<0.05; ** p<0.01; *** p<0.001.

Results confirmed that BMP9 was involved in pulpitis development. We assessed whether the recovery of the expression of BMP9 could affect the level of inflammation. In this case, BMP9 could positively affect the subsequent development of inflammation. After *Pg*-LPS stimulation, the expression of IL-6, IL-8, and CCL2 was upregulated after 3, 6, and 12 h (Figure 2 A–C), showing that the inflammatory microenvironment was successfully built. In the group with overexpressed BMP9, upregulated inflammatory genes were significantly downregulated at different time points (Figure 2 A–C). ELISA results showed that the protein level of IL-6 significantly reduced, in compliance with changes in gene expression (Figure 2 D). After *Pg*-LPS stimulation, the expression of MMP2 was significantly upregulated and BMP9 partially inhibited this expression (Figure 2 E and F). Besides detecting the effect of BMP9 alone on the inflammatory environment, we further evaluated the effect of BMP9 on the production of inflammatory cytokines by hDPCs in a non-inflammatory environment. Results showed that BMP9 also inhibited the expression of IL-6 and CCL2 in the normal cell culture environment (without *Pg*-LPS stimulation), but did not significantly regulate the expression of IL-8 (Supplementary Figure S2).

### Inflammatory environment inhibited by BMP9 in supernatant of hDPCs and reduced migration of THP-1 cells

**Figure 3:**
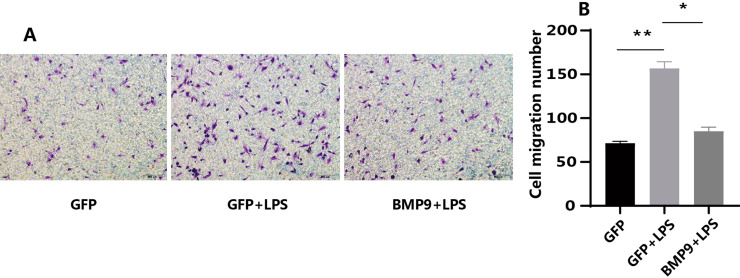
Effects of the supernatant of BMP9, which inhibited the inflammatory reaction of dental pulp cells in an inflammatory state, on the migration ability of THP-1 cells. (A) Crystal violet staining showed that the supernatant of hDPCs in the group treated with GFP+LPS promoted the migration of THP-1 cells while the supernatant in the group treated with BMP9+LPS inhibited this migration (transwell). (B) Quantitative analysis of crystal violet staining. Bars represent mean±SD (n=3). * p<0.05; ** p<0.01.

A previous study showed that BMP9 continuously inhibited the expression of CCL2. Therefore, we assessed whether the inflammatory environment inhibited by BMP9 could regulate the migration ability of monocyte-macrophages. The migration of THP-1 cells was induced after 24 h in the supernatants of groups treated with GFP, GFP+LPS, and BMP9+LPS. Crystal violet staining showed that compared with the group treated with GFP, the number of migrating THP-1 cells significantly increased in the supernatant of the group treated with GFP+LPS, whereas the THP-1 cell migration was inhibited in the supernatant of the group treated with BMP9+LPS (Figure 3 A and B).

### Involvement of the Smad1/5 and ERK/JNK-MAPK signaling pathways in BMP9-regulated *Pg*-LPS-induced inflammation of hDPCs

**Figure 4:**
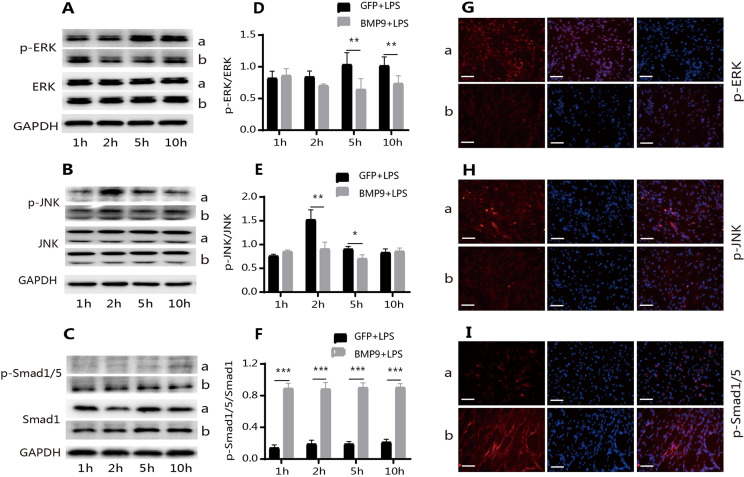
BMP9 increased the phosphorylation level of Smad1/5 and inhibited the phosphorylation level of ERK and JNK. WB showed that the overexpression of BMP9 inhibited the phosphorylation of ERK (A and D) and JNK (B and E) at 5 and 10 h and 2 and 5 h, respectively, and promoted the phosphorylation of Smad1/5 (C and F) in Pg-LPS-stimulated hDPCs. Immunocytofluorescence also showed that the overexpression of BMP9 inhibited the phosphorylation of ERK (G) and JNK (H) and increased the phosphorylation level of Smads1/5 (I). a: GFP+LPS; b: BMP9+LPS. Bars represent mean±SD (n=3). * p<0.05; ** p<0.01; *** p<0.001. scam bar: 75 μm.

After confirming that BMP9 inhibited inflammation, we evaluated the underlying mechanisms, identifying the signaling pathways most associated with BMP9 and the resulting inflammation. Smad1/5 and MAPK were found. Changes in NF-κB were also observed, but as they were insignificant, this study do not present these results. WB results suggested that after *Pg*-LPS stimulation, the phosphorylation levels of ERK and JNK were upregulated (Figure 4 A and D, and B and E) and the phosphorylation level of Smad1/5 did not change significantly (Figure 4 C and F). In the group treated with BMP9+LPS, the level of p-Smad1/5 significantly increased with time and was not affected by *Pg*-LPS (Figure 4 C and F). However, the level of p-ERK/JNK did not continuously increase, but significantly decreased (Figure 4 A and D, and B and E). Moreover, there was a lack of significant changes in p-p38 levels (Supplementary Figure S3). At the same time, changes in p-ERK/JNK and p-Smad1/5 levels were detected by immunofluorescence after 5 h and the results obtained were the same as those obtained using WB (Figure 4 G–I).

**Figure 5:**
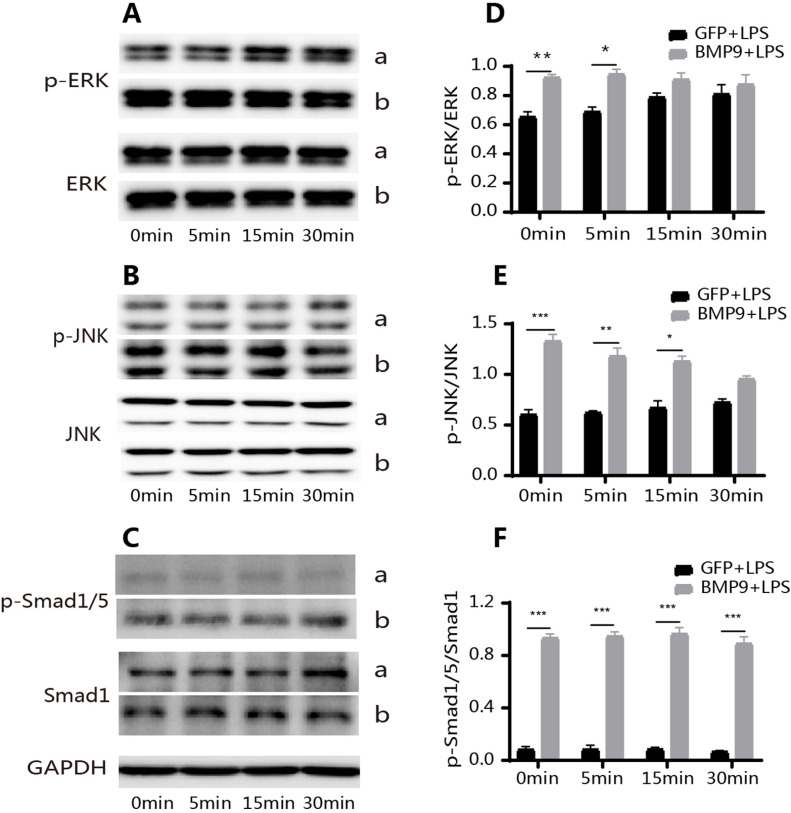
Effects of BMP9 on the phosphorylation level of Smad1/5, ERK, and JNK within 1 h. At 5, 15 and 30 min after Pg-LPS stimulation, the phosphorylation level of ERK (A and D) and JNK (B and E) in the group treated with BMP9+LPS gradually decreased. The phosphorylation level of ERK (A and D) and JNK (B and E) in the group treated with GFP+LPS gradually increased, but it was significantly lower compared with the group treated with BMP9+LPS (WB). Under the same conditions, the phosphorylation level of Smad1/5 (C and F) in the group treated with BMP9+LPS did not significantly change at different time points and was significantly higher compared with the group treated with GFP+LPS. The phosphorylation level of Smad1/5 (C and F) in the group treated with GFP+LPS was weak at different time points (WB). Bars represent mean±SD (n=3). * p<0.05; ** p<0.01; *** p<0.001.

Moreover, we found that the phosphorylation levels of ERK and JNK were slightly higher in the group treated with BMP9+LPS compared with the group treated with GFP+LPS after 1 h; thus, we assessed whether the same trend existed within the hour. The phosphorylation levels of ERK and JNK in the group treated with BMP9+LPS were significantly higher compared with the group treated with GFP+LPS 5, 15, and 30 min after the addition of *Pg*-LPS (Figure 5 A and D, and B and E). Under the same conditions, the expression of p-Smad1/5 in the group treated with BMP9+LPS was significantly higher compared with the group treated with GFP+LPS (Figure 5 C and F). The expression of P-smad1/5 was weak in the group treated with GFP+LPS and there was no significant change in the level of p-Smad1/5 between time points (Figure 5 C and F).

### Overexpressed BMP9 inhibited pulpitis *in vivo*

**Figure 6:**
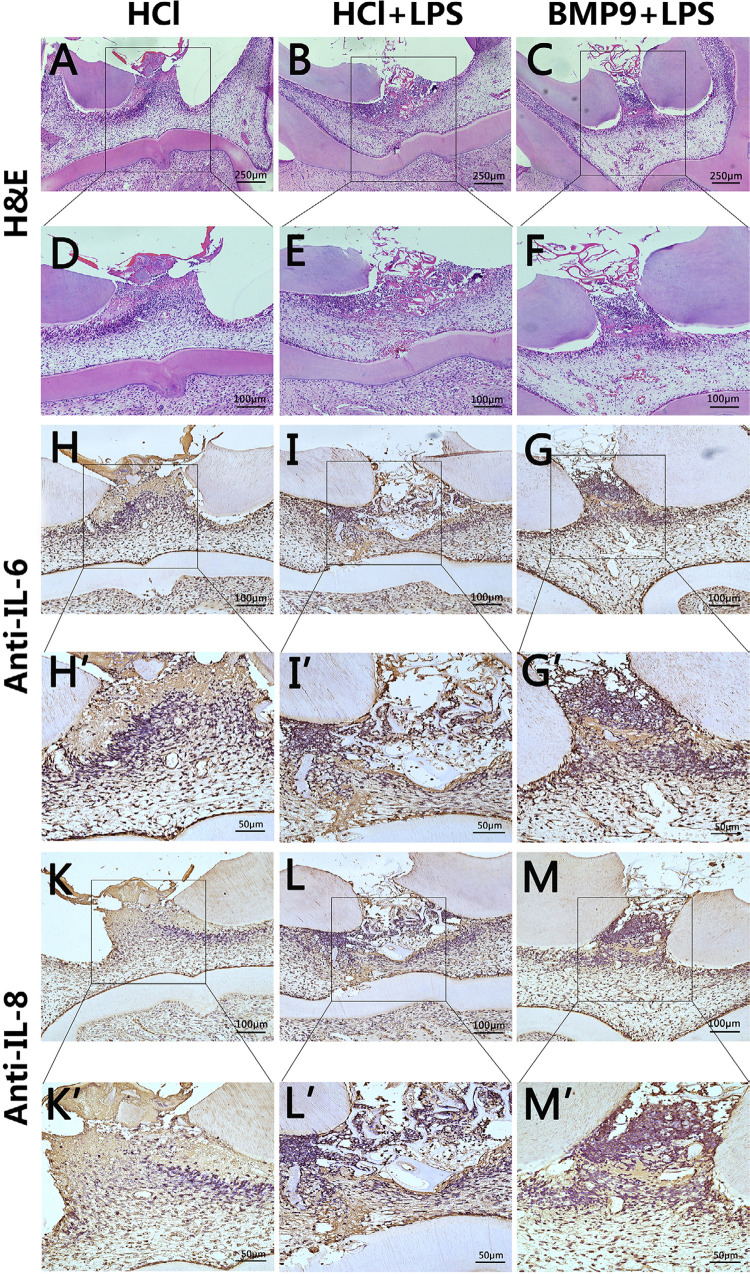
BMP9 inhibited the progression of early pulpitis in rats. (A–F) H&E showed the location and extent of pulpitis in the group treated with LPS+HCl and it was greater than in the other two groups. (H–M’) IHC results showed that IL-6 and IL-8 were mainly expressed in the inflammatory region and the expression range and level of IL-6 and IL-8 in the group treated with LPS+HCl were higher compared with the other two groups.

To further evaluate the effect of BMP9 on pulpitis *in vivo*, we used direct rat pulp capping models. To overexpress BMP9, 1 mg/mL of the BMP9 recombinant protein was used in this experiment. Previous studies with animals showed that the expression of BMP9 decreased after 24 h; therefore, we chose the initial stage of inflammation as our observation point. Rats were euthanized for sample collection 24 h after treatment. H&E staining showed that the inflammatory area was concentrated below the opened pulp. The degree of inflammation and the amount of inflammatory cell infiltration in the group treated with LPS+HCl were significantly higher compared with the group treated with LPS+BMP9 or HCl (Figure 6 A–F). The inflammatory areas in the group treated with LPS+BMP9 were similar to those in the group treated with HCl, but there were more inflammatory cells (Figure 6 A and D, and C and F). The expression of IL-6 and IL-8 were evaluated in the groups treated with LPS+HCl and LPS+BMP9. Compared with the group treated with LPS+HCl, more limited ranges and lower expression of IL-6 and IL-8 were found in the group treated with LPS+BMP9 (Figure 6 H–M’).

## Discussion

Finding biological factors that can effectively regulate pulpitis and promote pulp tissue repair is important to VPT. BMP9 may have this function. Our previous study confirmed that BMP9 could promote pulp tissue repair *in vivo* and *in vitro*. In the affected area, inflammation is present; therefore, this study further evaluated the regulatory effect of BMP9 on pulpitis and the relationship between BMP9 and pulp tissue repair. BMP9 has attracted much attention due to its unique properties and roles in tooth development and odontogenic differentiation.^11,12^ Several studies also assessed the link between BMP9 and inflammation.^24,25^

This study showed that BMP9 was involved in the regulation of the occurrence and development of pulpitis. Using a rat tooth model, we successfully induced chronic pulpitis, which was confined to the coronal pulp on day 3. In mature rat pulp tissue, our results showed that BMP9 was mainly expressed in the odontoblast cell layer and the remaining pulp cells, in accordance with our previous results regarding human pulp tissue.^13^ The expression of BMP9 significantly decreased in the inflammatory area and surrounding odontoblast layer 24 h after the onset of inflammation, showing that the expression of BMP9 was inhibited in the early stage of pulpitis. After early suppression, BMP9 recovered its expression after 3 d. Similar results were found in hDPCs stimulated with *Pg*-LPS *in vitro*. This phenomenon was similar to the results of previous studies, showing that BMP9 was associated with trauma or LPS-induced acute inflammation.^24^ After intraperitoneal injection of LPS or partial liver resection, the expression of BMP9 in the liver tissue and blood of rats sharply decreased after 3 h. The expression of BMP9 gradually returned to normal or became higher than the normal values after about 18 h (American Thoracic Society International Conference, 2018). Although times were different, the trend was the same. The expression of BMP9 decreased in the early stage of inflammation in different tissues, showing the close relationship between BMP9 and inflammation. We will continue to evaluate the specific mechanisms of the suppressed expression of BMP9 in future studies. We also found that BMP9 was significantly overexpressed in the inflammatory marginal area on days 3 and 7 of pulpitis. Thus, we assessed whether BMP9 suppressed the inflammatory response in this case.

LPS is a toxic product of inflammation caused by gram-negative bacteria and causes many infectious bacterial diseases. It is widely used *in vitro* to stimulate cells to form an inflammatory environment.^26^ In a short period after *Pg*-LPS stimulation, DPCs secrete abundant inflammatory factors, such as IL-6, IL-8, and IL-1β, and the CCL2 chemokine.^27^ IL-1β is highly produced in inflammatory pulp tissue; however, there is controversy on whether IL-1β is expressed after LPS stimulation of DPCs.^28^ IL-6 and IL-8 are key elements involved in inflammation and their expression in the pulpitis region is high. IL-8 is a chemotactic factor for neutrophils and CCL2 is a chemotactic factor for macrophages, which are important participants in the inflammatory process. Therefore, IL-6, IL-8, and CCL2 were chosen in this study as key targets to assess the level of inflammation. BMP9 significantly reduced the expression of IL-6, IL-8, and CCL2 produced by *Pg*-LPS-stimulated hDPCs after 3, 6, and 12 h. The regulatory effect of BMP9 on inflammatory cytokines has also been reported. The secretion of CCL2 was inhibited in both human vascular endothelial cells and pulmonary arterial endothelial cells treated with BMP9, in accordance with our results.^29^ Recent studies showed similar results, since BMP9 inhibited the expression of IL-6 and IL-8 to prevent cancer cell metastasis.^15,16^ However, a higher concentration (5 ng/mL) of BMP9 made LPS stimulate the secretion of IL-6 and IL-8 by vascular endothelial cells, contrary to the results of our study.^24^ This may be mainly due to different cell sources and concentrations of BMP9, as the biological role of BMP9 varies depending on its concentration and context. Thus, further research is needed. Moreover, data from another study from our team showed that BMP9 promoted the expression of inflammatory factors in human periodontal ligament stem cells after 3 d or more.^30^ Different terminal cells and stages of inflammation (early and later) may account for the difference between results, showing once more the complexity of BMP9 and inflammation, which we will further evaluate. MMP2 is an important extracellular matrix degradation enzyme and inflammatory mediator that is significantly upregulated in pulpitis.^31^ Studies found active MMP2 only in inflamed pulps.^32^ MMP2 in the inflammatory region can participate in the inflammatory process by degrading the gelatin and different types of collagen. The effect of BMP9 on the expression of MMP2 in *Pg*-LPS-stimulated hDPCs was evaluated again and results showed that *Pg*-LPS significantly increased the expression of MMP2 whereas BMP9 partially inhibited the upregulation of MMP2, which was in line with the inhibitory effect of BMP9 on inflammatory factors, suggesting the inhibitory role of BMP9 in inflammation.

It is believed that the signal transduction of BMP9 is mainly performed by Smad1/5-dependent and non-Smad-dependent pathways (MAPKs) of BMP9.^33^ However, pathogen infection and tissue damage can also activate the MAPK signaling pathway, which mediates the development of inflammation.^34^ Our results showed that when inhibiting *Pg*-LPS-induced inflammation, BMP9 activated Smad1/5 and inhibited the phosphorylation levels of ERK and JNK. In contrast, the phosphorylation level of p38 did not significantly change. BMP9 activates classic Smad1/5 in all circumstances and MAPKs were activated in most studies, especially on osteogenesis, but this phenomenon changed in the inflammatory context. For example, as aforementioned, BMP9 inhibits the metastasis of cancer cells under an inflammatory state by inhibiting MAPKs and Akt or MAPK/ERK and NF-κB, which is similar to our results. It shows that the effect of BMP9 on MAPKs varies in response to inflammation. Moreover, after inflammation, the phosphorylation levels of ERK and JNK regulated by BMP9 changed from high to low. MAPKs, as an important component of inflammatory activation pathways, are controlled in the presence of BMP9. Another interesting finding was that p-p38 did not change significantly at the point set by our study; however, this result was not surprising because all three MAPKs do not always function simultaneously.^35,36^ Immune cells can kill bacteria and remove necrotic material, which makes them conducive to inflammation control, but they can also accidentally injure normal tissues, resulting in tissue damage. In osteoarthritis, the CCL2/CCR2 signaling axis can induce monocytes to be recruited to inflammatory areas, worsening inflammation and tissue damage.^37^ Our results showed that inflammatory responses in the supernatant of hDPCs were inhibited by BMP9, reducing the migration ability of monocytes-macrophages and suggesting that the inhibition of inflammation by BMP9 may be harmful to the chemotaxis of immune cells.

In our direct rat pulp capping model, pulp tissue inflammation could have been caused by the dual effects of ball drill heat generation and *Pg*-LPS stimulation. The inflammation of dental pulp tissue was clearer after the addition of *Pg*-LPS compared with the ball drill heat-producing group alone, including the increased amount of immune cell infiltration and increased expression of inflammatory factors, which also showed that *Pg*-LPS triggered the generation of dental pulp tissue inflammation *in vivo*, in accordance with previous results.^19^ The level of inflammation in groups treated with LPS+BMP9 was in line with *in vitro* results, but weaker compared with the group treated with LPS+HCl, which shows that BMP9 has an inhibitory effect on early pulpitis. However, the intensity of inflammation in early stages has an important effect on the overall development of inflammation. In several *in vivo* studies on the liver, the expression of BMP9 decreased with stimulation time (which we consider to be the early or acute stage of inflammation), which is similar to our results. However, there is still a lack of studies on the mechanisms of downregulation of the expression of BMP9. Moreover, the effects of the overexpression of BMP9 on the development of inflammation during this period are unknown. Therefore, our results showed that in the early stage of inflammation, BMP9 might regulate inflammation by inhibiting the expression of inflammatory cytokines. Further studies are needed to assess the role of BMP9 in the later stage of inflammation. However, in the long-term pulp capping experiment, our research team observed that BMP9 promoted a significant pulp tissue repair, which seems to indirectly suggest the regulation of BMP9 on the long-term inflammatory response.

## Conclusion

This study showed that BMP9 is involved in the occurrence and development of pulpitis. The expression of BMP9 decreased in the early stage of inflammation and recovered later. Moreover, the overexpression of BMP9 might inhibit inflammation in the early stage of pulpitis by the Smad1/5 and ERK/JNK-MAPK pathways. This study, combined with our previous research, showed that BMP9 might be a potential regulatory factor of pulpitis and promote pulp tissue repair, which provides a reference for the study of bioactive molecule materials in VPT.
